# Ecological risk assessment of future suitable areas for *Piper kadsura* under the background of climate change

**DOI:** 10.3389/fpls.2024.1471706

**Published:** 2025-01-20

**Authors:** Shimeng Li, Yuanxin Li, Mingli Hu, Yankun Li, Mingrong Yang, Shi Wang, Wei Yu, Chunsong Cheng, Qiqing Cheng

**Affiliations:** ^1^ School of Pharmacy, Xianning Medical College, Hubei University of Science and Technology, Xianning, Hubei, China; ^2^ Hubei Engineering Research Center of Traditional Chinese Medicine of South Hubei Province, Xianning, Hubei, China; ^3^ Faculty of Chinese Medicine and State Key Laboratory of Quality Research in Chinese Medicine, Macau University of Science and Technology, Macau, Macao SAR, China; ^4^ Lushan Botanical Garden, Chinese Academic of Sciences, Jiujiang, Jiangxi, China

**Keywords:** *Piper kadsura*, environmental variable, habitat suitability, species distribution, ArcGIS

## Abstract

**Introduction:**

*Piper kadsura* is a well-known medicinal plant that belongs to woody liana, possessing high therapeutic and economic value. The market demand of *P. kadsura* is huge, but its wild resources are scarce and artificial cultivation methods have not been established, which leads to a situation with strong contradiction and imbalance between supply and demand.

**Methods:**

In this study, 303 sample of distribution data for *P. kadsura* in China were collected, 33 environmental variables related to terrain, climate and soil were analyzed and the suitable habitats of *P. kadsura* during various periods were predicted by MaxEnt model and ArcGIS software, aiming to provide a basis for scientific cultivation and effective utilization of resources.

**Results:**

The results indicated that precipitation and temperature were significant factors in the distribution of *P. kadsura*. The primary environmental variables influencing the potential distribution of *P. kadsura* were precipitation during the driest quarter (Bio17), annual precipitation (Bio12), mean diurnal range (Bio2), and annual temperature range (Bio7). Among them, precipitation of driest quarter (Bio17) was the most influential environmental variable for the distribution of *P. kadsura* with the range between 100.68 and 274.48 mm. The current distribution of *P. kadsura* is mainly located in the coastal areas of eastern and southern China, especially Guangxi, Guangdong, Zhejiang and Fujian, with a total area of 51.74 × 104 km2. Future climate change of global warming will lead to a reduction in the total suitable areas and high suitable areas under various climate scenarios. Especially in the SSP585 scenario, the total suitable area and the highly suitable area will be significantly reduced by 89.26% and 87.95% compared with the present during the 2090s.

**Discussion:**

Overall, these findings can provide useful references for the suitable areas’ determination of wild resources, optimization of artificial cultivation and scientific selection of high quality medicinal materials on *P. kadsura*.

## 1 Introduction

The global climate is continuously changing, impacting the Earth’s weather system in various ways, including seasonal patterns, extreme and unexpected weather events, temperature fluctuations, and changes in precipitation (Kunwar et al., 2023). The intensification of global warming, driven by human activities and natural disasters, is anticipated to result in a higher frequency and severity of climate change in the future (Wang Y. et al., 2024). Climate is a crucial factor influencing species distribution (Hou Z. et al., 2023) and plants are particularly sensitive to climate change, which may lead to the migration of plant habitats and alterations in their suitable areas (Duan et al., 2022; Nanda et al., 2021). Over time, the rates of shifts in species distribution, habitat loss and fragmentation, as well as species extinctions, are expected to increase *(* Subedi et al., 2024). Currently, many Chinese medicinal materials are primarily sourced from wild resources, which typically possess significant medicinal value, such as *Pellionia scabra* (Chen T. et al., 2022), *Gentiana rhodantha* (Zhang et al., 2022), *Rheum nanum* (Xu et al., 2022), and others. Current habitats that support wild populations may become unsuitable in the future due to rapidly changing climate conditions (Adhikari et al., 2023). Assessing the distributional changes of medicinal plants in relation to bioclimatic variables can provide valuable insights to specific variables that significantly influence their distribution (Subedi et al., 2023). This information is crucial for the proactive planning of protected areas, for warning against potential extinction events (Fan et al., 2022) and for offering guidance on the conservation, development, utilization, and artificial cultivation of medicinal plant resources. Currently, the application of climate data to construct species distribution models has been widely applied in the study of suitable habitats for plants (Sun et al., 2023; Yang et al., 2023). Several models are commonly used to analyze the potential suitable habitats of species, including genetic algorithm for rule set production (GARP), bioclimatic analysis and prediction system (BIOCLIM), random forests (RF), general additive model (GAM), general linear model (GLM), generalized boosting model (GBM), artificial neural network (ANN), multiple adaptive regression splines (MARS), and maximum entropy (MaxEnt) (Varela et al., 2011; Li and Wang, 2013; Melo-Merino et al., 2020). These models can comprehensively consider various environmental variables, including climate, terrain and soil, to accurately predict the potential distribution areas of species. Among them, the MaxEnt model constructs and predicts species distribution by calculating the probability distribution of maximum entropy, accurately identifying key variables affecting species distribution in complex environmental conditions (Cao et al., 2021). This model stands out due to its advantages such as requiring fewer samples, being less influenced by sample variation and providing precise predictions. It has been widely applied in various fields, including conservation biology and ecology (Kumar et al., 2022; Shen et al., 2023).

*Piper kadsura* is a vine-like medicinal plant found mostly in the littoral regions of southern China (Liu et al., 2015). The stem part of *P. kadsura* is a traditional Chinese medicine called “haifengteng”. It serves as a key ingredient in the classical prescriptions of Juanbi decoction and Gunan-Yizhi decoction. These prescriptions have been widely used for the treatment of gout, rheumatoid arthritis and vascular dementia (Wang et al., 2020; Hu et al., 2022). According to the modern chemical and pharmacological studies, *P. kadsura* mainly comprises the compounds of terpenes, amide alkaloids and neolignans, which having the effects of anti-neuroinflammation, anti-oxidation and anti-inflammatory (Huang et al., 2021; Chen H. et al., 2022). A recent study shows that futoquinol from *P. kadsura* has the activity of nerve cell protection and is a potential drug for the treatment of Alzheimer’s disease (Zhang et al., 2024). The stems of *P. kadsura* have medicinal properties, while the entire plant is utilized as a food source. It is recognized as an important medicinal and edible plant, characterized by its extensive applications and significant potential market demand (Kim et al., 2010). Due to climate change and the thermophilic habit of *Piper* genus plants, the origins of *P. kadsura* gradually moved south from Qinling Mountains to coastal areas. Because *P. kadsura* is the only species source of “haifengteng” in all the versions of Chinese Pharmacopoeia, and the rapid growth of *P. kadsura* consumption and the current situation of resources highly dependent on the wild sources (Lee et al., 2016; Meng et al., 2023). However, the distribution area of *P. kadsura* is very limited at present, forming a situation with strong contradiction and imbalance between supply and demand.

Currently, there are few reports on the potential suitable areas for *P. kadsura*. This study is the first to conduct research on the potential suitable areas for *P. kadsura*. We collected and organized the distribution data of *P. kadsura*, combined it with three environmental factors: climate, soil, and terrain. The MaxEnt model and ArcGIS software were used for modeling to analyze the potential suitable areas for *P. kadsura* in the past (LGM, MH), present (1970-2000) and future (2050s, 2090s). This study has two objectives: (1) to evaluate the current distribution of *P. kadsura* and the factors influencing it and (2) to investigate how the range of this species may change under future climate scenarios. The distribution of *P. kadsura* is predominantly concentrated in the coastal regions of southern China. We hypothesize that its distribution will decline as a result of climate change.

## 2 Materials and methods

### 2.1 Acquisition and screening of distribution data

**Figure 1:**
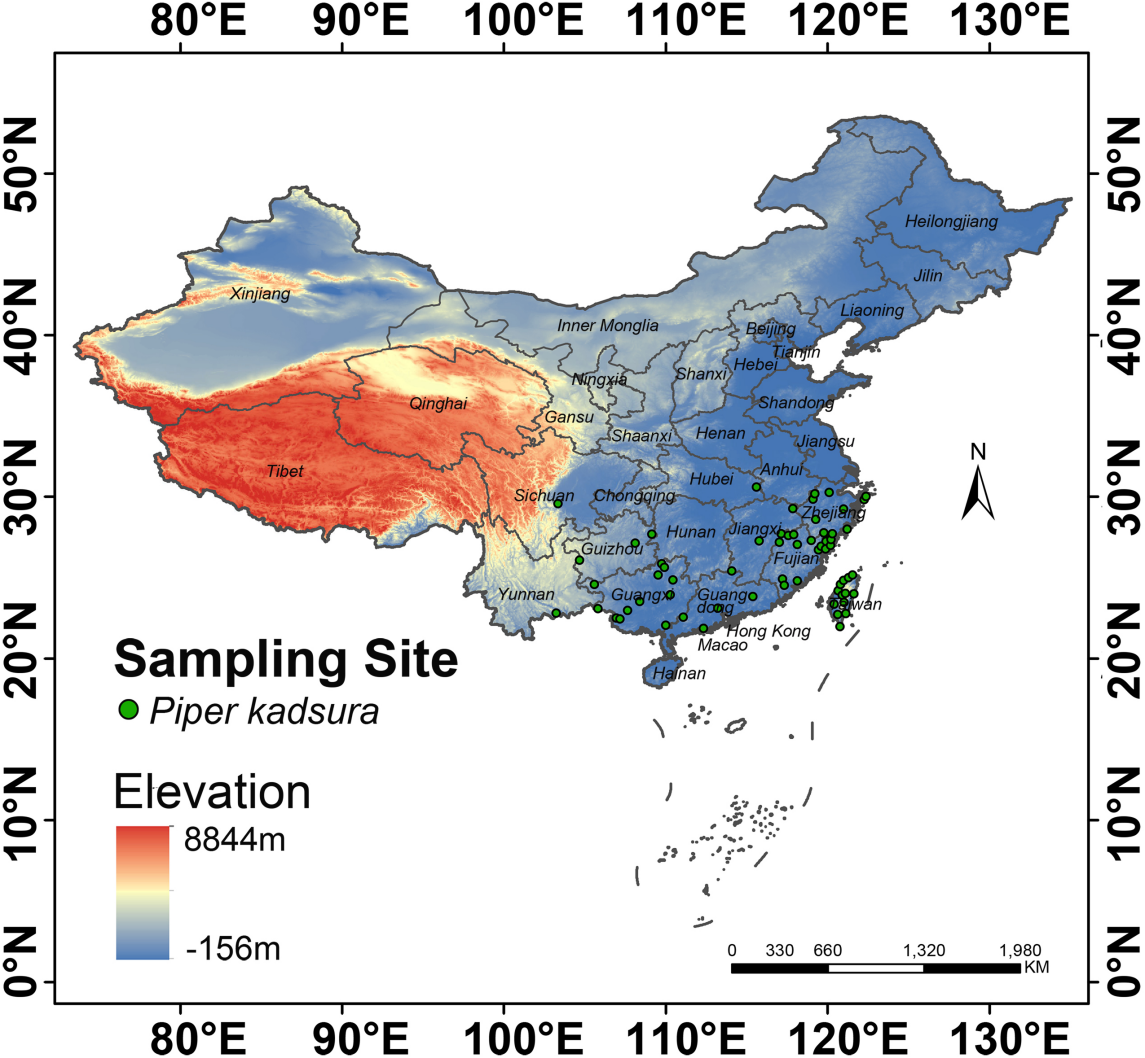
Distribution map of *P. kadsura* in China.

By consulting online databases such as the Chinese Virtual Herbarium (http://www.cvh.ac.cn/) and the NSII-China National Specimen Resource Platform (http://www.nsii.org.cn/), a total of 303 records were collected nationwide and their distribution information was also obtained (**Supplementary Table S1**). Then, duplicate coordinate data and samples with unclear geographic distribution location information were removed. According to the reported method (Wu et al., 2024), the coordinate information of sample points with clear geographical locations was determined using baidu coordinate picker (https://api.map.baidu.com/lbsapi/getpoint/index.html), and 89 samples of *P. kadsura* were finally obtained (**Supplementary Table S2**). When utilizing this tool, if the provided location information was not accurate beyond the district or county level, automatic identification of coordinates became unattainable. In such cases, manual positioning was required. When manually locating an area, it was crucial to confine within the boundaries of the county. Otherwise, there might be significant deviations. Additionally, to reduce model overfitting caused by sampling bias, neighborhood analysis in ArcGIS 10.4.1 was used to set a buffer zone with a radius of 10 km, and one distribution point was randomly retained within a range of 20 km, eventually resulting in 65 valid distribution points (**Supplementary Table S3**; **Figure 1**). The species name, longitude, and latitude of these points were applied for subsequent analysis (Zhang et al., 2019).

### 2.2 Acquisition and screening of environmental variables

Nineteen climate variables were obtained from the World Climate Database (http://www.worldclim.org) using current (1970-2000) climate data as the baseline, selecting the Last Glacial Maximum (LGM) and the Mid-Holocene (MH) for past climate data, as well as different scenarios for future climate (2041-2060, 2081-2100). Future climate data were determined based on the Shared Socioeconomic Pathways (SSPs) models released by the Sixth Coupled Model Intercomparison Project (CMIP6), with SSP126 (low emission scenario) and SSP585 (high emission scenario) reflecting the most optimistic and pessimistic greenhouse gas emission scenarios for the future, respectively (Riahi et al., 2017). Meanwhile, eleven soil variables and three topographic variables were obtained from the Food and Agriculture Organization of the United Nations World Soil Database (http://www.fao.org/soils-portal/data-hub/en/) and the WorldClim website (https://www.worldclim.org/) (Fan et al., 2022; Ouyang et al., 2022). A total of 33 environmental variables were applied to evaluate the impact on distribution of *P. kadsura*, and the most dominant environmental variables were found out after eliminating strongly correlation variables that are relatively minor (**Table 1**).

**Table 1:** Description of environmental data.

Variable	Description	Variable	Description
Bio1	Annual mean temperature	Bio18	Precipitation of warmest quarter
Bio2	Mean diurnal range (mean of monthly (max temp - min temp))	Bio19	Precipitation of coldest quarter
Bio3	Isothermality (bio2/bio7) (× 100)	awc_class	Soil available water content
Bio4	Temperature seasonality (standard deviation × 100)	s_caco3	Topsoil calcium Carbonate
Bio5	Max temperature of warmest month	s_clay	Substrate-soil clay content
Bio6	Min temperature of coldest month	s_oc	Substrate-soil organic carbon
Bio7	Temperature annual range (bio5-bio6)	s_ph_h2o	Substrate-soil pH
Bio8	Mean temperature of wettest quarter	s_sand	Sediment content in the subsoil
Bio9	Mean temperature of driest quarter	t_caco3	Topsoil carbonate or lime content
Bio10	Mean temperature of warmest quarter	t_clay	Clay content in the upper soil
Bio11	Mean temperature of coldest quarter	t_oc	Topsoil organic carbon
Bio12	Annual precipitation	t_ph_h2o	Topsoil pH
Bio13	Precipitation of wettest month	t_sand	Sand content
Bio14	Precipitation of driest month	aspect	Aspect
Bio15	Precipitation seasonality (coefficient of variation)	elev	Elevation
Bio16	Precipitation of wettest quarter	slope	Slope
Bio17	Precipitation of driest quarter		

In order to reduce the high correlation and multicollinearity among environmental variables that cause model overfitting and ensure the accuracy of the prediction, this study used SPSS 26.0 software to perform Spearman correlation analysis on the above environmental variables (**Supplementary Figure S1**). When the correlation coefficient of two environmental variables was greater than |0.8|, the variables with small contribution rates were eliminated, thus minimizing the bias fitting of the MaxEnt mode (Yang et al., 2022). Ultimately, 18 environmental variables were retained to construct the prediction model for *P. kadsura*, including 8 climatic variables (Bio17, Bio12, Bio7, Bio2, Bio3, Bio18, Bio1, Bio15), 7 soil variables (t_ph_h2o, s_clay, s_oc, t_sand, s_caco3, s_ph_h2o, t_oc), and 3 topographic variables (aspect, elev, slope).

### 2.3 Construction of the MaxEnt model

The distribution data of *P. kadsura* and effective environmental variables were imported into the MaxEnt software (V3.4.3) to predict its potential suitable habitat distribution. The following modeling parameters were used: sampling method was bootstrap, output format was logistic, and 75% of the distribution points were randomly selected as the training set, with the remaining 25% of the distribution points as the test set. For each training partition, after 106 iterations and 10 times model repetition, the average value of the calculations was taken as the final result of the model prediction (Huang et al., 2023). This study selected the area under the Receiver Operating Characteristic (ROC) curve (AUC) to evaluate the accuracy of the model prediction (Xu et al., 2020). At the same time, the Jackknife method was used to analyze the impact of each environmental variable on the distribution of *P. kadsura* and to plot the response curves of key environmental variables. In addition, in species distribution modeling, the Maximum Test Sensitivity Plus Specificity Logistic Threshold (MTSPS) was used as the dividing line between suitable and unsuitable areas, which is considered simple and effective (Aidoo et al., 2022; Huang R. et al., 2022). With reference to the methods of Yang et al. (2023) and Wu et al. (2024), we used MTSPS to classify their potential suitable habitats into the following four levels the MTSPS was used to divide its potential suitable habitat into the following four levels: unsuitable habitat (0~MTSPS), low suitability habitat (MTSPS~0.3), medium suitability habitat (0.3~0.5), and high suitability habitat (0.5~1), and the area of different suitability habitats was calculated (Yan et al., 2020).

## 3 Results and analysis

### 3.1 Model accuracy analysis

**Figure 2:**
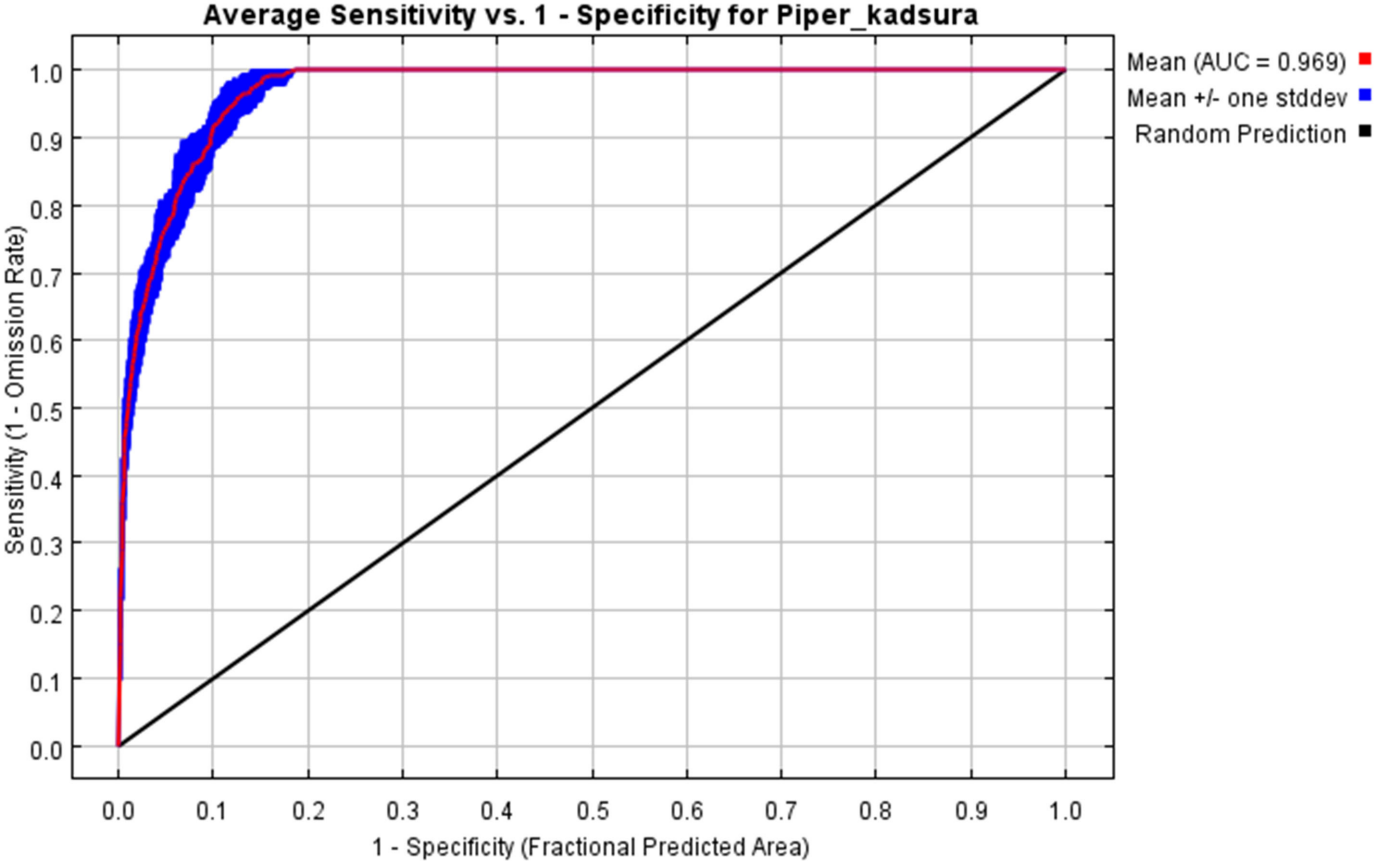
ROC curve of the MaxEnt model.

By simulating and predicting the distribution area of the *P. kadsura* through MaxEnt software, the average ROC curve of 10 calculation results was finally obtained after 10 loops. The AUC value ranged from 0 to 1, and the closer it approached 1, the more accurate the prediction result of the model was. When AUC was more than 0.9, the model prediction was excellent (Ouyang et al., 2022). As shown in **Figure 2**, the average training value of the ROC curve in this study was 0.969, indicating that the construction of this model had a very high accuracy and could be used to study the potential suitable habitat of the *P. kadsura*.

### 3.2 Analysis and selection of key environmental variables

To characterize the effects of various environmental variables on the construction results of the prediction model, we used the MaxEnt model to analyze the contribution rates and permutation importance of 18 environmental variables separately. The percent contribution represented the percentage of the impact of climatic factor on the model after all variables are considered, while permutation importance indicated the degree of impact on the model after the factor has been replaced (Hou J. et al., 2023). As shown in **Table 2**, Bio17 (precipitation of driest quarter) had the highest contribution rate at 52.0%, followed by Bio12 (annual precipitation) at 21.9%. The contribution rates of Bio7 (temperature annual range), slope, Bio2 (mean diurnal range), Bio3 (isothermality), aspect, and elev (elevation) were 5.8%, 4.5%, 2.8%, 2.8%, 2.6%, and 2.1% respectively. The contribution rates of the remaining environmental variables were all below 2.0%. Among them, Bio2, Bio7, and elev had relatively high confidence importance values of 38.8%, 17.1%, and 10.7% respectively, indicating a strong dependence of the model on these three variables (Wang E. et al., 2024).

**Table 2:** Percent contribution and permutation importance of dominant environmental variables of the MaxEnt model.

Variable	Description	Percent contribution (%)	Permutation importance (%)
Bio17	Precipitation of driest quarter	52.0	5.7
Bio12	Annual precipitation	21.9	3.7
Bio7	Temperature annual range (bio5-bio6)	5.8	17.1
slope	Slope	4.5	4.0
Bio2	Mean diurnal range (mean of monthly (max temp - min temp))	2.8	38.8
Bio3	Isothermality (bio2/bio7) (× 100)	2.8	8.1
aspect	Aspect	2.6	0.5
elev	Elevation	2.1	10.7
Bio18	Precipitation of warmest quarter	1.8	4.6
t_ph_h2o	Topsoil pH	0.6	0.2
s_clay	Substrate-soil clay content	0.6	0.3
Bio1	Annual mean temperature	0.6	3.2
Bio15	Precipitation seasonality (coefficient of variation)	0.5	1.0
s_oc	Substrate-soil organic carbon	0.4	0.3
t_sand	Sand content	0.4	0.6
s_caco3	Topsoil calcium Carbonate	0.4	0.5
s_ph_h2o	Substrate-soil pH	0.2	0.2
t_oc	Topsoil organic carbon	0.1	0.3

**Figure 3:**
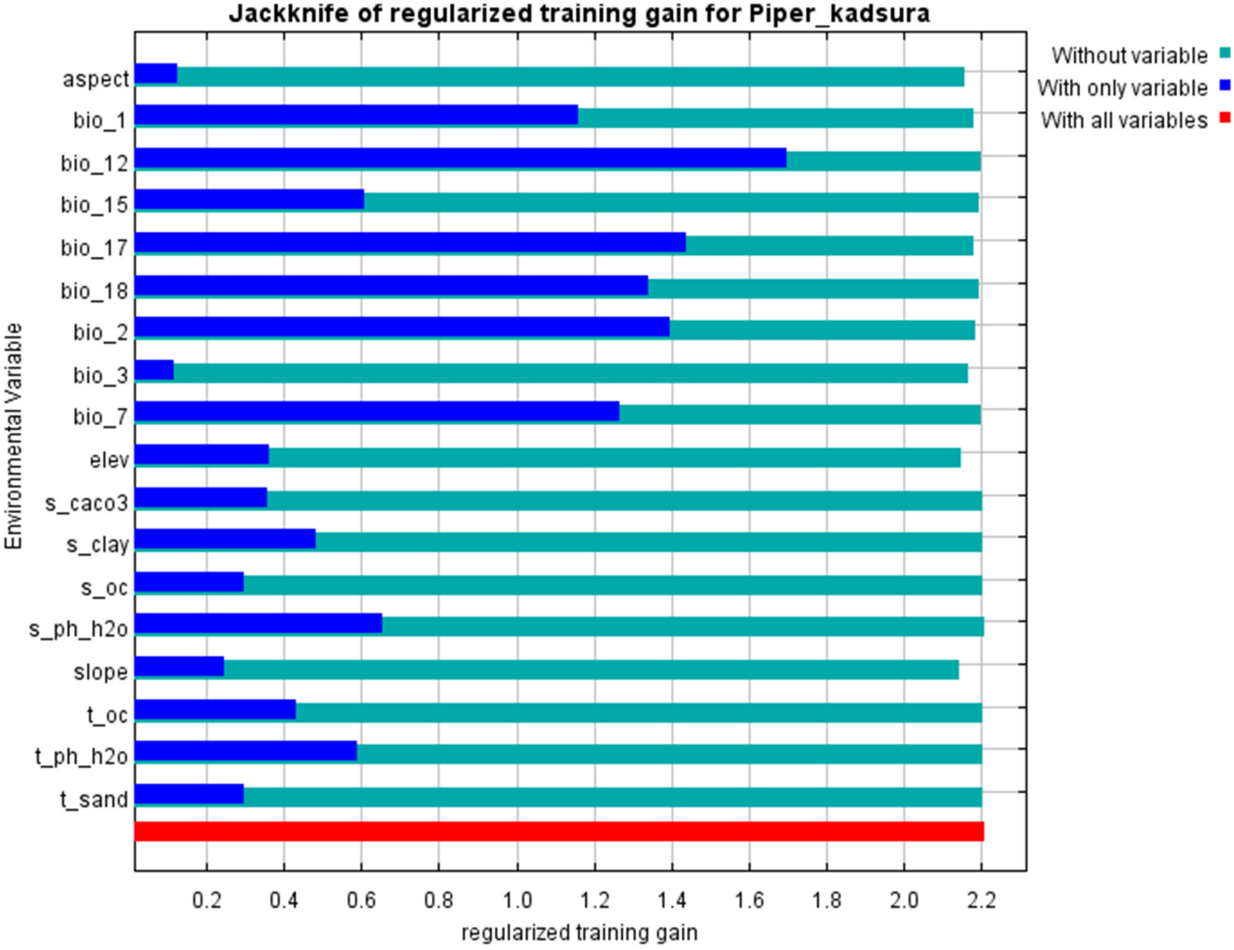
Jackknife test of environmental variables for *P. kadsura*.

To characterize the importance of various environmental variables on the distribution of *P. kadsura*, we used the jackknife test to examine the impact of dominant environmental factors on the suitable distribution area of *P. kadsura* in China (**Figure 3**). The results indicated that Bio12, Bio17, and Bio2 had the greatest impact on the distribution of *P. kadsura*, suggesting that these three environmental variables contain more effective information compared to others (Deng et al., 2024). On the whole, the dominant environmental variables influencing the distribution of *P. kadsura* were precipitation of Bio17, Bio12, Bio2, and Bio7. Therefore, it could be inferred that temperature and precipitation are key factors affecting the distribution of *P. kadsura*.

**Figure 4:**
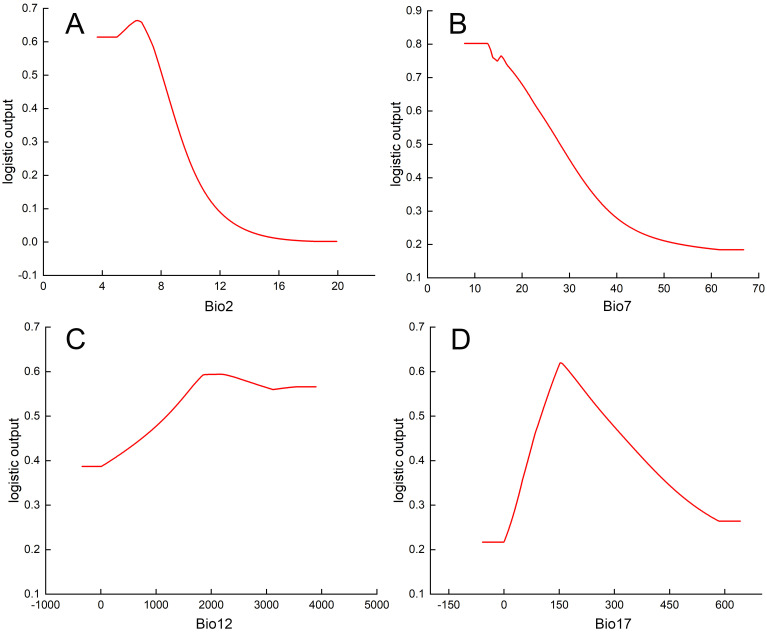
Response curves of key influencing factors. **(A)** Mean diurnal range, Bio2 (°C); **(B)** Temperature annual range, Bio7 (°C); **(C)** Annual precipitation, Bio12 (mm); **(D)** Precipitation of driest quarter, Bio17 (mm).

Then, based on the response curves of the key environmental variables derived above, the relationship between the distribution probability of *P. kadsura* and the environmental variables can be determined. When the distribution probability of *P. kadsura* was greater than 0.5, the corresponding environmental variable values were favorable for the growth of *P. kadsura*. The response curves (**Figures 4A–D**) showed that the value ranges (and optimal values) of the key environmental variables that limited the distribution of *P. kadsura* were: Bio17 100.68-274.48 mm (153.24 mm), Bio12 1194.10-3898.20 mm (2190.12 mm), Bio7 7.82-28.00°C (12.66°C), Bio2 3.65-8.06°C (6.42°C). The distribution probability raised with the increase in the values of key environmental variables before the optimal values, and decreased with the increase in the environmental factor values after the optimal values.

### 3.3 Distributional projections in the current climate

**Figure 5:**
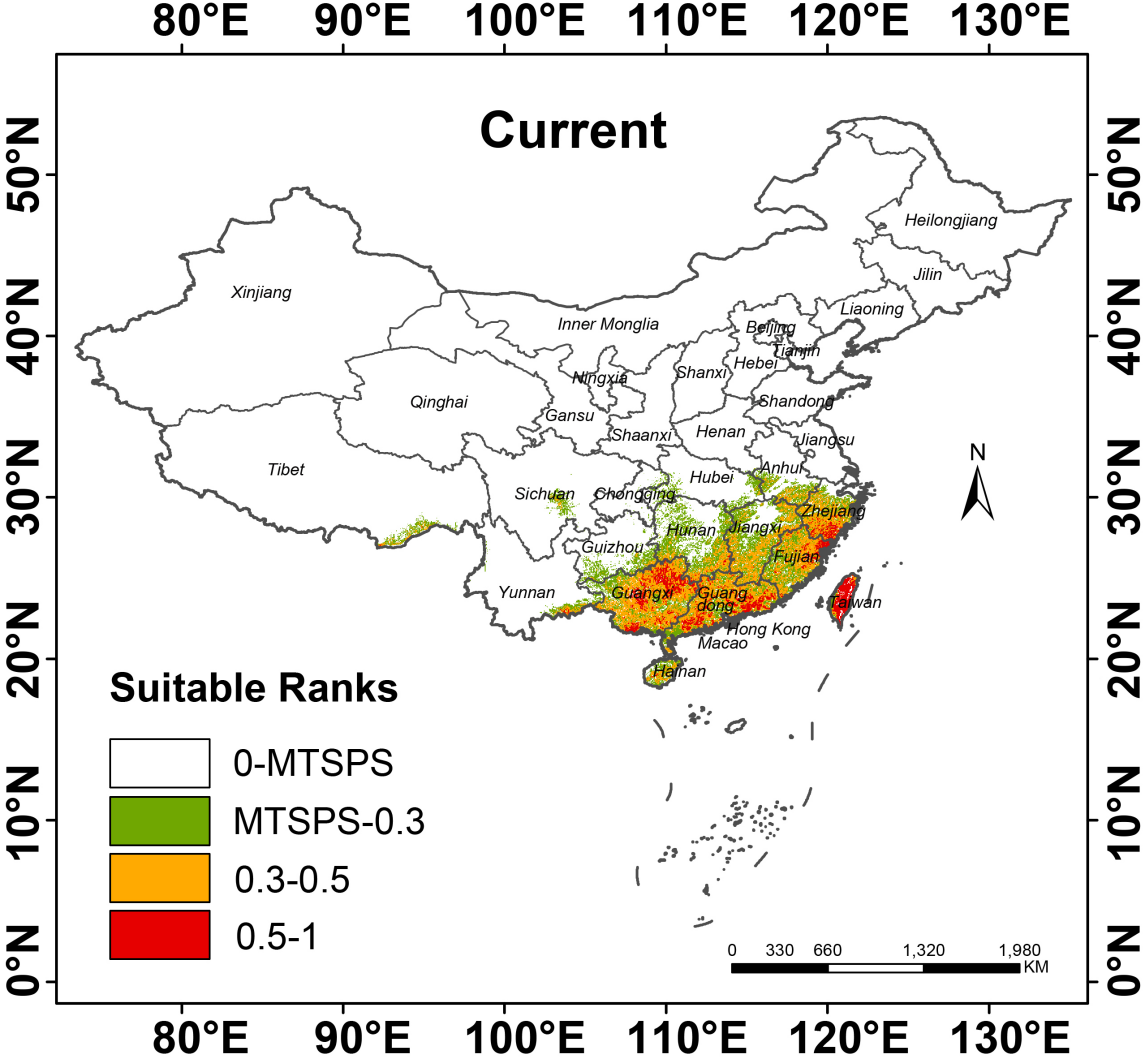
Distribution of suitable habitats for *P. kadsura* under current scenario.

According to the “Flora Reipulicae Popularis Sinicae”, *P. kadsura* was distributed along the coastal areas of China, especially in the provinces of Fujian and Zhejiang, growing in low-altitude forests, climbing on trees or rocks (Flora of China Editorial Committee of Chinese Academy of Sciences, 1982). The distribution records in the NSII-China National Specimen Resource Platform showed that *P. kadsura* is mainly distributed in Guangxi (129 distribution points), Guangdong (94 distribution points), Fujian (77 distribution points), Zhejiang (69 distribution points), Guizhou (42 distribution points), Taiwan (35 distribution points), Yunnan (22 distribution points), Sichuan (16 distribution points), Jiangxi (15 distribution points), and Hainan (12 distribution points), with sporadic distribution in other provinces. As shown in **Figure 5**, the white areas represented unsuitable zones of *P. kadsura*, the green areas represented low suitability zones, the yellow areas represented medium suitability zones, and red represents high suitability zones. The main distribution range of *P. kadsura* was between 105° E - 121° E and 18° N - 30° N, including medium and high suitability zones, with a total suitable area of 51.74 × 10^4^ km², accounting for 5.39% of China’s land surface area, while the high suitability zone was accounting for only 22.32% of the total suitable area (**Table 3**). Currently, the distribution of the total suitable area for *P. kadsura* was relatively concentrated, mainly located in the coastal areas of East and South China, with rare distribution in inland regions, and none as it moved further north, which was highly consistent with the natural distribution area recorded in the “Flora Reipulicae Popularis Sinicae”. Among them, the high suitability zones were mainly distributed in Taiwan, Guangxi, Guangdong, Zhejiang, and Fujian provinces. The medium suitability zones were distributed around the high suitability zones, mainly covering Jiangxi, Hainan, southern Anhui, southern Hunan, southeastern Guizhou, and southeastern Yunnan. The low suitability zone area was 38.49 × 10^4^ km², accounting for 4.01% of China’s land surface area (**Table 3**). The unsuitable zones were mostly located in the northern and southwestern regions of China, with large areas in Henan, Hubei, northern Jiangsu, and northern Anhui.

**Table 3:** Statistical analysis of suitable areas of *P. kadsura* in different periods.

period		Unsuitable habitat		Low suitability habitat		Medium suitability habitat		High suitability habitat	
		Area (×10^4^ km^2^)	Percent-age (%)	Area (×10^4^ km^2^)	Percent-age (%)	Area (×10^4^ km^2^)	Percent-age (%)	Area (×10^4^ km^2^)	Percent-age (%)
LGM		960	100	0	0	0	0	0	0
MH		960	100	0	0	0	0	0	0
Current		869.77	90.60	38.49	4.01	40.19	4.19	11.55	1.20
2050S	SSP126	914.30	95.24	31.67	3.30	11.50	1.20	2.53	0.26
	SSP585	895.70	93.30	43.53	4.53	17.87	1.86	2.90	0.30
2090S	SSP126	868.09	90.43	42.43	4.42	39.63	4.13	9.85	1.03
	SSP585	935.79	97.48	18.65	1.94	4.17	0.43	1.39	0.14

The area percentages represented the ratio of each suitable area to the land surface area of China (960 × 10^4^ km^2^) in each period.

### 3.4 Prediction of distribution under past and future climates

**Figure 6:**
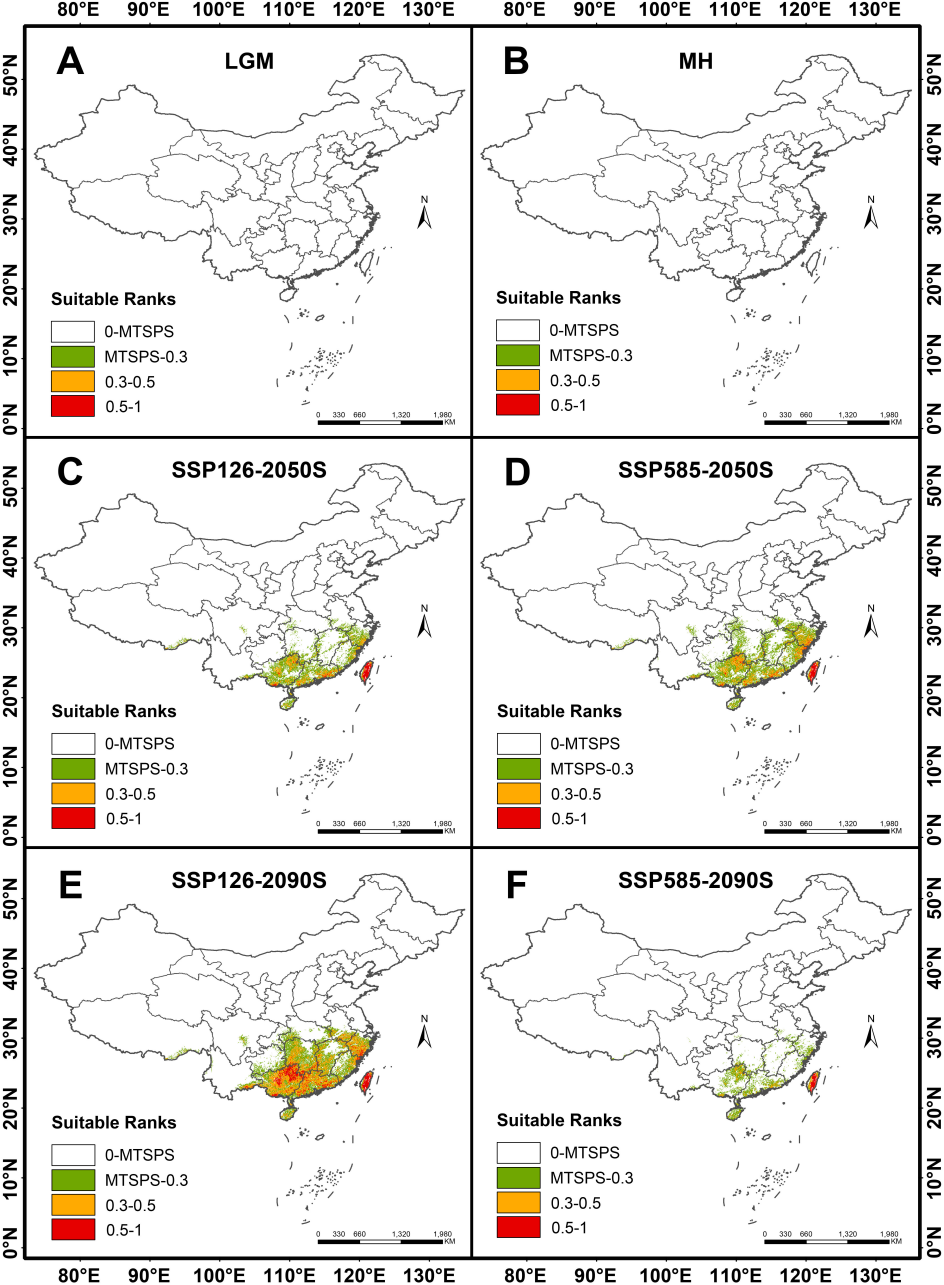
Distribution of suitable habitats for *P. kadsura* under different climate scenarios. **(A)** Last Glacial Maximum (LGM); **(B)** Mid-Holocene (MH); **(C)** Average for 2041-2060 (2050S), SSP126; **(D)** Average for 2041-2060 (2050S), SSP585; **(E)** Average for 2081-2100 (2090S), SSP126; **(F)** Average for 2081-2100 (2090S), SSP585.

This study selected 6 periods to predict the potential distribution of *P. kadsura* in China. Based on the prediction results of the MaxEnt model, habitat suitability distribution maps of *P. kadsura* under two scenarios (SSP126, SSP585) in the 2050s and 2090s were obtained. As shown in **Figures 6A, B**, from the Last Glacial Maximum (LGM) to the Mid-Holocene (MH), *P. kadsura* had no suitable habitat, with a total suitable area of 0. From MH to the present, the suitable area increased to the maximum, with the current total suitable area being 51.74 × 10^4^ km^2^. The high suitable area covered 11.55 × 10^4^ km^2^, accounting for 1.20% of China’s land surface area (**Table 3**). This indicated that the current climate was more suitable for the survival of *P. kadsura*, while during the LGM and MH periods, *P. kadsura* could not survive, which might be related to the cold climate during the LGM and the unstable climate during the MH period.

**Figure 7:**
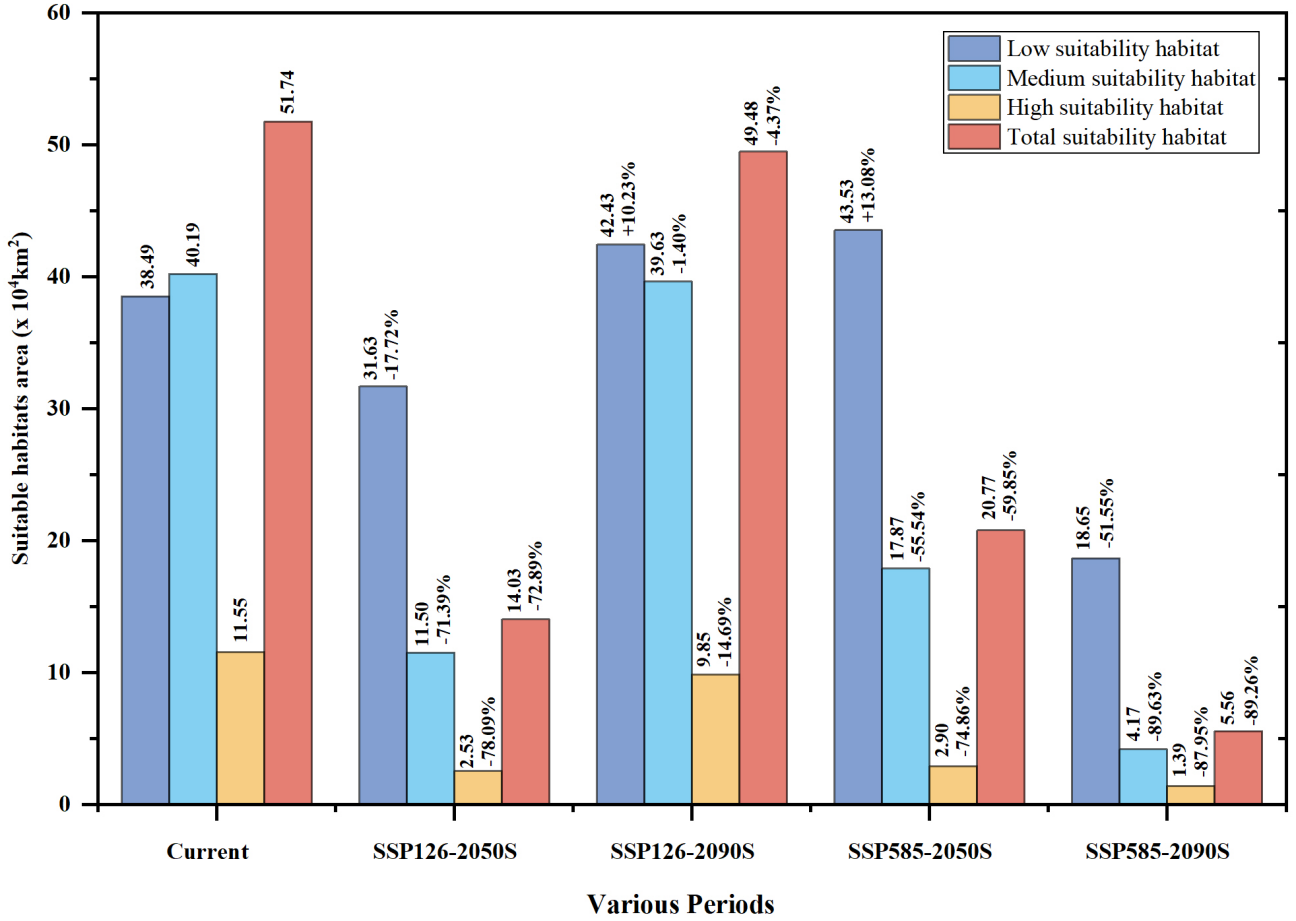
Percentage of suitable area change of *P. kadsura* under future climate compared to current climate. At the top of the columns, the numbers on the left represented the potential distribution area of *P. kadsura* in different periods; the numbers on the right represented the proportion of change in distribution area compared to the current climate, with "+" and“-” indicating the percentage increase and decrease in potential distribution area.

From the present to the future, the distribution range of *P. kadsura* would be reduced to varying degrees, showing a trend of initial decrease followed by an increase under the SSP126 scenario. However, compared to the current distribution range, there would still be a certain degree of reduction (**Figures 6C–F**). The future suitable habitats of *P. kadsura* shrinking and expanding were shown in **Table 3** and **Figure 7**. Under the SSP126 scenario, the total suitable area from 2041 to 2060 was 14.03 × 10^4^ km^2^, a decrease of 72.89% compared to the current climate scenario. The high suitable area decreased by 78.09%, while the low and medium suitable areas decreased by 17.72% and 71.39%, respectively. From 2081 to 2100, the total suitable area was 49.48 × 10^4^ km^2^, a decrease of 4.37% compared to the current climate scenario. The low suitable area increased by 10.32%, while the high and medium suitable areas decreased by 14.69% and 1.40%, respectively. Under the SSP585 scenario, the total suitable area from 2041 to 2060 was 20.77 × 10^4^ km^2^, a decrease of 59.85% compared to the current climate scenario. The low suitable area increased by 13.08%, while the high and medium suitable areas decreased by 74.86% and 55.54%, respectively. From 2081 to 2100, the total suitable area was 5.56 × 10^4^ km^2^, a decrease of 89.26% compared to the current climate scenario. The high, medium, and low suitable areas decreased by 87.95%, 89.63%, and 51.55%, respectively.

## 4 Discussion

### 4.1 Key environmental variables affecting the distribution of suitable habitats

Current and future climatic scenarios were modeled for the potential distribution of high-value and low-yield medicinal plant *P. kadsura* in the coastal areas of China. Precipitation contributed the most to the model scores, up to 73.9%, of which the precipitation of driest quarter (Bio17) accounted for 52%, and the annual precipitation (Bio12) accounted for 21.9%. Following was the temperature range that contributed 8.6% to the prediction scores, of which temperature annual range (Bio7) accounted for 5.8%, and the mean diurnal range (Bio2) accounted for 2.8%. These high-contribution variables were similar to those for *Eremochloa ophiuroides* (Xu et al., 2024). Specifically, *P. kadsura* maintained good growth efficiency when Bio17 was between 100.68-274.48 mm, Bio12 was between 1194.10-3898.20 mm, Bio7 was between 7.82-28.00°C, and Bio2 was between 3.65-8.06°C. Plants generally required sufficient water to meet transpiration needs and maintain normal physiological functions. When some researchers cultivated *P. kadsura*m, the precipitation of the selected locations as 1750.00-1800.00 mm (Jiang, 2017) and 1662.00 mm (Li, 2015), which was in line with the predicted range of 1194.10-3898.20 mm for Bio12. Additionally, the geographic distribution of *P. kadsura* was mainly in the coastal areas of eastern and southern China, which were characterized by a subtropical monsoon climate with distinct seasons and warm, humid conditions, also aligned well with the prediction results.

Besides precipitation, temperature played a significant role in the formation and distribution of plants. With the increase of temperature, the stomatal opening on the plant surface enlarged, the plant transpiration and respiratory rate increased significantly, and eventually lead to substantial losses of water, thereby inhibiting growth (Zhu et al., 2023). The primary temperature influence factors for *P. kadsura* were the Bio7 and Bio2, achieving optimal growth efficiency at 7.82-28.00°C and 3.65-8.06°C, respectively. This indicated that *P. kadsura* was not suitable for areas with large temperature differences and was not found in northern regions with significant temperature variations. Moreover, appropriate diurnal temperature variation can promote plant growth (Sun et al., 2000). In this study, the suitable value of Bio2 for the *P. kadsura* growth was 3.65-8.06°C, and exceeding this range was detrimental to its growth. Furthermore, numerous studies have also shown that precipitation and temperature were crucial variables affecting species distribution. For instance, Chang et al. (2020) studied the impact of climate change on the potential distribution of *Anabasis aphylla* in Northwestern China. Li et al. (2022) predicted the potential suitable areas for *Glycyrrhiza uralensis*, and Jiang et al. (2023) explored the potential suitable areas for *Panicum milliaceum* under climate change, all concluded that temperature and precipitation were major factors influencing the potential distribution of plants.

Therefore, in the future protection and cultivation of *P. kadsura*, the influence of temperature and precipitation should be fully considered. The results of this study can provide information for the suitable habitat of *P. kadsura*, but further practical exploration and summary are needed for subsequent practical applications.

### 4.2 Impact of climate change on the suitable habitat of *P. kadsura* and resource conservation

As global climate continues to be warm and intensified, accompanied by frequent extreme events, the suitable habitats distribution of many species will be reduced, and the habitat fragmentation will be serious (Barbarossa et al., 2021). Sudden changes in the living environment will affect the migratory ability of species. If a species has weak migratory ability and its distribution speed is slower than the rate of climate change, it will not be able to adapt to the climate change quickly, making it easy for sensitive and ecologically poorly adaptable species to decline in distribution or become extinct (Zhang et al., 2019). Therefore, understanding the distribution of species’ survival under climate change is of great importance for assessing the impact of climate change on species and formulating conservation measures. Additionally, genetic diversity of species should also be fully considered. Potential suitable habitat simulations during different periods indicate that climate change significantly affects the species. The predicted results of this study show that *P. kadsura* did not have any distribution during the LGM and MH periods, possibly because the LGM was the most recent extremely cold period, with approximately 24% of the global land covered by ice and frequent extreme cold events (Zhan et al., 2022), which did not meet the survival conditions of *P. kadsura*. During the MH period, the climate was warmer and more humid than the present, with significant climate fluctuations. *P. kadsura* is a perennial vine, the instability and abrupt change of climate had a great impact on its growth in the next year, making this period also unsuitable for its survival. From the MH period to the present, *P. kadsura* transitioned from no distribution to having the largest total suitable habitat area, indicating that the current climate conditions favor the growth of *P. kadsura*.

Compared to the present, the future high-temperature environment caused by carbon emissions showed an overall shrinking trend in the suitable habitat of *P. kadsura*. Under the low-emission SSP126 scenario, the future suitable habitat area of *P. kadsura* fluctuates significantly, with a sharp reduction in 2050S, shrinking by 72.89% compared to the current total suitable habitat area, and generally retreating to the southeastern coastal areas. This indicated that the environmental conditions during this period were not suitable for the growth of *P. kadsura*. In this scenario, the distribution of *P. kadsura* in the 2090S period was more optimistic than in the 2050S period but still reduced compared to the present. The total suitable habitat area was only reduced by 4.35% compared to the present, with an insignificant reduction degree, but the high suitability area was reduced by 14.69%. This indicated that the climate conditions in the high suitability area during this period did not provide a better growth environment for *P. kadsura* compared to the present, thus limiting its growth. Under the high-emission SSP585 scenario, the suitable habitat area of *P. kadsura* shrunk rapidly in the future, with a reduction of 89.25% by the 2090S period compared to the present. This indicated that under the high-emission scenario, *P. kadsura* cannot adapt to the changing climate environment, experiencing severe growth limitations, and might face endangerment and extinction in the future. The study by He and Ding (2023) showed that compared to the high-emission scenario, the low-emission scenario had a greater possibility of reducing future climate risks. In the face of climate change, the distribution of suitable habitats for plants will respond to varying degrees. However, in the context of continuous warming, the future distribution of *P. kadsura* is unfavorable, with a reduction in total suitable habitat area. This is consistent with research findings for *Alternanthera philoxeroides* (Yan et al., 2020), *Dipteronia sinensis* (Huang Y. et al., 2022) and *Entodon challenger* (Cong et al., 2023).

### 4.3 Limitations and prospects for this study

Many researches only use climate variables to predict suitable habitats for species, this study applies climate (Zhang et al., 2021; Zuo et al., 2022), soil and terrain as environmental variables, which can improve the accuracy of the suitable habitats prediction for *P. kadsura*. However, there are many models developed and ensembled to analyze potential suitable habitats. Kunwar et al. (2023) used ensemble model to predict the distribution of seven medicinal plant species of Nepal. Subedi et al. (2023) predicted the distribution of the endangered Maple Leaf oak (*Quercus acerifolia*) using an integrated model. MaxEnt model stands out for our study because it requires fewer samples and has the advantages of accurate prediction. The prediction of our model may not exactly match actual developments, because only a single MaxEnt model is applied, and the SSP model is based on assumptions of future conditions, rather than direct observation. In addition, model predictions alone are not sufficient to confirm claims about the evolutionary history or origin of *P. kadsura*. In the following study, we will try to use more models, increase sample quantity, apply R language method, and analyze actual distribution point samples to improve the accuracy of prediction. And consider incorporating phylogenetic evidence or fossil data to strengthen the inferences about the past distributions and evolutionary history of *P. kadsura*. However, it is important to note that despite some limitations, this study still provide reference value for the sustainable development and utilization of *P. kadsura*, as well as add literature support for applying species distribution models to assess the effects of climate change on the future distribution of species.

According to the above analysis, we need to implement protective measures for the resources with high-value and low-yield resources. First of all, for the areas that having distribution records or areas identified as high suitability, it is essential to clarify their specific geographic locations and growth patterns, carry out the continuous monitoring about the surrounding habitat and growth conditions, and strengthen the personalized protection of the environmental conditions. Furthermore, it’s essential to identify suitable locations for cultivation and conservation, and then conduct the necessary transplantation, cultivation, and breeding activities to establish a strong foundation for the responsible development and exploitation of *P. kadsura*.

## 5 Conclusion

*P. kadsura* is an important medicinal plant in China, but the shrinking suitable habitats lead to the serious imbalance in demand and resources. Our findings indicated *P. kadsura* will still face an obvious decrease in habitat suitability under different climate scenarios in the future. The suitable area of *P. kadsura* will gradually shrink to the southern coastal areas of China, in which precipitation and temperature range were the key environmental variables affecting the suitable habitats area. A predicted loss of more than 70% of current habitat was predicted by 2050 under the low-emission scenario, and even nearly 90% loss of suitable habitat is predicted by 2090 under the highest greenhouse gas emission scenario. *P. kadsura* will experience extreme vulnerability due to climate change. The large geographic shifts projected under very low to extreme climate change scenarios constitute a major threat for *P. kadsura* survival. And the restoration of degraded planting areas within high suitable habitats is essential for the sustainable protection of the *P. kadsura*. Our analysis contributes to the prediction of future distribution of *P. kadsura*, a precious medicinal plant with high-value and low-yield, and can be utilized as a valuable management and conservation planning basis for this important species.

## Data Availability

The raw data supporting the conclusions of this article will be made available by the authors, without undue reservation.
