# Supplementary material for: Ecological risk assessment of future suitable areas for Piper kadsura under the background of climate change
Source: Front Plant Sci. 2025 Jan 20;15:1471706. doi: 10.3389/fpls.2024.1471706 (PMC11788358; doi:10.3389/fpls.2024.1471706)
Supplement: Supplementary file 1 [file SupplementaryFile1.docx]

Supplementary Material

# Supplementary Figures and Tables

## Supplementary Tables

Supplementary tables has been uploaded separately at the time of submission.

**Supplementary Table 1.** Distribution information of all *Piper kadsura* points collected in this study (303 samples).

**Supplementary Table 2.** Distribution information of *Piper kadsura* excluding the duplicate points and unknown information points (89 samples).

**Supplementary Table 3.** Distribution information of *Piper kadsura* excluding overlapping points within 10km (65 samples).

## Supplementary Figures


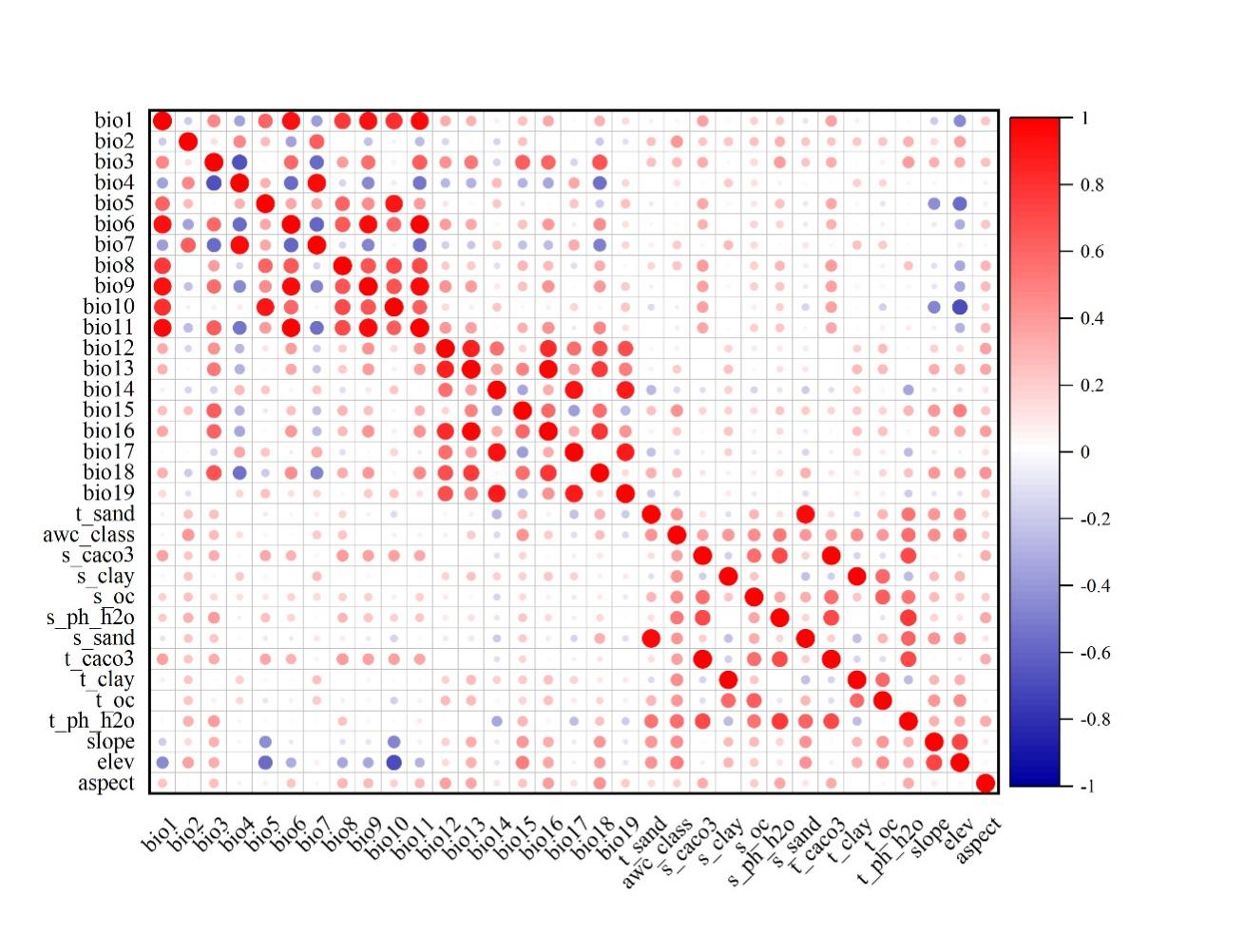


**Supplementary Figure 1.** Correlation of environmental variables.
